# Supplementary material for: Shared genetic factors between osteoarthritis and cardiovascular disease may underlie common etiology
Source: Nat Commun. 2024 Nov 6;15:9569. doi: 10.1038/s41467-024-53812-2 (PMC11538479; doi:10.1038/s41467-024-53812-2)
Supplement: Supplementary file 1 — Supplementary Information [file 41467_2024_53812_MOESM1_ESM.pdf]

Supplementary Appendix for the paper

Shared genetic factors between osteoarthritis and cardiovascular disease may underlie common etiology

by Magnusson et al., 2024

Contents:

S-Table 1:	Included OA and CVD outcomes.	p. 2
S-Table 2:	Measurement of physical activity level.	p. 2
S-Figure 1:	The bivariate twin model with example.	p. 3
S-Table 3:	Sex-specific concordance for each outcome.	p. 4
S-Table 4:	Model fit for main models.	p. 5
S-Table 5:	Sex differences in the association between any-site OA and any of CVDs.	p. 5
S-Table 6:	Sex differences in the association between any-site OA and any of CVDs.	p. 5
S-Table 7:	Number of pairs concordant for both OA and CVDs, among pairs in which both twins survived through the entire follow-up period.	p. 6
S-Table 8:	Correlations across traits, among pairs in which both twins survived through the entire follow-up period.	p. 6
S-Methods 1	Computer code for bivariate ACE model with two binary outcomes	p. 7
S-Methods 2	Computer code for Cox regression analysis of cumulative concordance for OA and severe CVD	p. 12

S-Table 1. Included outcomes		
Outcome	***Category***	***ICD-10 diagnostic code***
Osteoarthritis	Hip	M16
	Knee	M17
	Hand	M15.1, M15.2, M19.0D- M19.2D
Severe CVD	Cardiac arrythmias	I47, I48, I49
	Coronary heart disease (CHD), including acute myocardial infarction, valvular disease and angina pectoris	I20, I21, I22, I23, I24, I25, I34, I35, I36, I37
	Heart failure	I50
	Stroke (hemorrhagic and ischemic)	G45, G46, I60, I61, I62, I63, I65, I66, I69

S-Table 2. Measurement of physical activity level		
Year	***Question***	***Response***
1967	"How much exercise have you performed when you were aged 25-50 years?"	Categories: 1) almost none, 3) light exercise e.g. walking or guardening, 5) regular exercise through sports, 7) hard/heavy exercise. Alternatives 2, 4, 6 and 8 were available responses but not described in words, yet most likely, a higher number reflect a higher amount of exercise.
1967	"Own physical activity"	Categories: 0) Else, 1) almost no exercise
1970	"Own physical activity"	Categories: 0) Else, 1) almost no exercise
1973	"Physical activity during leisure time"	Categories: 1) Almost no exercise, 2) Hardly any exercise, 3) Very little exercise, 4) Little exercise, 5) Between little and much exercise, 6) Much exercise, 7) Very much exercise.
2000	Not measured	
2006	"Indicate your current physical activity on a scale from 1 to 10, from very low to very high."	Categories: From 1) Very low, to 10) Very high
2012	"How much do you move on an average day? The question refers to physical activity at home, at work, as well as exercise, walking, cycling, etc"	Categories: 1) Mostly sitting, 2) Sitting and walking a little, 3) Walking around 30min per day, 4) Moving and exerting myself moderately, 5) Strenous activity at least 60min per day.

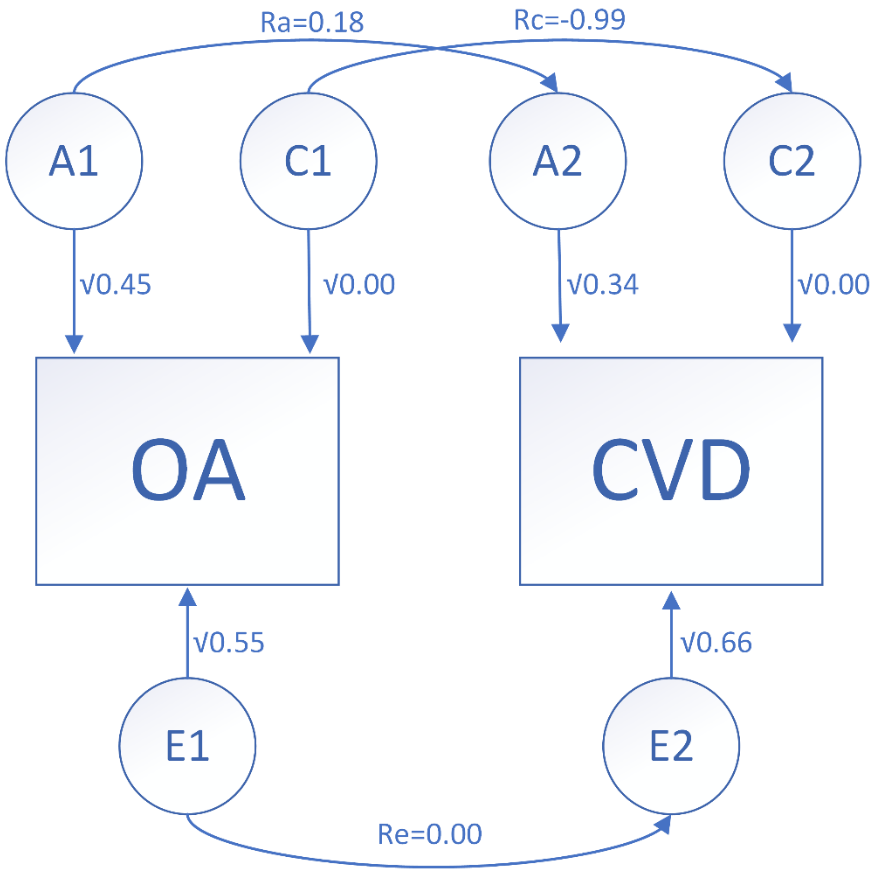

S-Figure 1. The bivariate twin model showing the parts of the variance explained by additive genetic factors (A), common environmental factors (C), and unique environmental factors (E). Ra, Rc and Re represent the correlations that are due to these respective factors. Example of parameter estimates are from the model including any site OA and any of severe CVDs, adjusted for age, sex and BMI, on 29 985 twin pairs, where 145 had any site OA (hip, knee and/or hand OA) and any of CVDs The phenotypic correlation (rPT) due to genetic effects (A) can be calculated based on individual heritabilities for each trait (√A1 and √A2) and the genetic correlation between these traits (Ra), i.e. [√A1 * Ra * √A2)/rPT]. Estimates for √C1, √C2 and Re are different from zero, more exactly 0.00000000007734912, 0.000000004155556 and 0.001517876, respectively.

S-Table 3. Sex-specific concordance for each outcome.

		Members of pairs concordant for two traits						
	All	Any-site OA and any of CVDs	Any-site OA and card. Arr.	Any-site OA and CHD	Any-site OA and heart failure	Any-site OA and stroke	Hip OA and any of CVDs	Knee OA and any of CVDs
Total nr of pairs	N=29 985	N=145	N=39	N=49	N=15	N=18	N=38	N=44
Same sex MZ twins, male	4128	34	2	9	1	4	7	5
Same sex DZ twins, male	5400	27	12	14	5	3	9	14
Same sex MZ twins, female	5347	30	7	12	1	3	4	10
Same sex DZ twins, female	6617	26	13	9	6	3	9	8
Opposite sex DZ twins	8493	28	5	5	2	5	9	7

S-Table 4. Model fit for all models in main analysis.				
Outcome	OA site	Model	BIC	AIC
Any CVD	Any	Crude	106221.5	106165.0
Any CVD	Any	Age+sex	96076.78	95999.84
Any CVD	Any	Age+sex+BMI	09448.35	90372.67
Any CVD	Knee	Crude	90663.06	90606.63
Any CVD	Knee	Age+sex	80895.72	80818.78
Any CVD	Knee	Age+sex+BMI	67116.73	76091.05
Any CVD	Hip	Crude	82639.01	82582.58
Any CVD	Hip	Age+sex	72668.58	72591.64
Any CVD	Hip	Age+sex+BMI	68739.75	68664.07
Card. Arr	Any	Crude	77098.29	77041.86
Card. Arr	Any	Age+sex	74047.84	73970.90
CHD	Any	Crude	84494.75	84438.33
CHD	Any	Age+sex	79296.06	79219.12
Heart fail	Any	Crude	65761.68	65761.68
Heart fail	Any	Age+sex	61399.02	61322.08
Stroke	Any	Crude	76752.31	76695.88
Stroke	Any	Age+sex	71740.78	71663.84

S-Table 5. Sex differences in the association between any-site OA and any of CVDs.

	Males and females		Males and females	
	MZ males and MZ females N=9475	DZ opposite-sex N=8492	MZ males and MZ females N=9475	DZ same-sex N=12 018
Number of concordant pairs (percent of total)*	64 (0.68)	28 (0.33)	64 (0.68)	53 (0.44)
Crude rPT (95% CI)	0.18 (0.16-0.20)		0.16 (0.14-0.18)	
Crude xtxt r (95% CI)	0.18 (0.15-0.21)	0.11 (0.09-0.14)	0.16 (0.13-0.19)	0.09 (0.07-0.12)
Adjusted rPT (95% CI)	0.09 (0.05-0.11)		0.09 (0.07-0.12)	
Adjusted xtxt r (95% CI)	0.08 (0.05-0.11)	0.04 (0.03-0.06)	0.09 (0.07-0.12)	0.08 (0.05-0.11)

S-Table 6. Sex differences in the association between any-site OA and any of CVDs.

	Males only		Females only	
	MZ males N= 4128	DZ males N=5401	MZ females N=5347	DZ females N= 6617
Number of concordant pairs (percent of total)*	34 (0.82)	27 (0.50)	30 (0.56)	26 (0.39)
Crude rPT (95% CI)	0.21 (0.17-0.24)		0.13 (0.10-0.16)	
Crude xtxt r (95% CI)	0.19 (0.15-0.23)	0.10 (0.07-0.13)	0.14 (0.11-0.18)	0.10 (0.06-0.13)
Adjusted rPT (95% CI)	0.14 (0.11-0.17)		0.06 (0.03-0.09)	
Adjusted xtxt r (95% CI)	0.10 (0.05-0.14)	0.05 (0.03-0.07)	0.07 (0.03-0.11)	0.04 (0.02-0.07)

S-Table 7. Number of pairs concordant for both OA and CVDs, among pairs in which both twins survived through the entire follow-up period.

	Any site OA		Knee OA		Hip OA	
	MZ conc. pairs (% of all MZ twins)	DZ conc. pairs (% of all MZ twins)	MZ conc. pairs (% of all MZ twins)	DZ conc. pairs (% of all MZ twins)	MZ conc. pairs (% of all MZ twins)	DZ conc. pairs (% of all MZ twins)
Any of CVDs	26 (0.37)	32 (0.23)	10 (0.14)	9 (0.06)	6 (0.09)	7 (0.05)
Card. Arr.	14 (0.20)	6 (0.04)	5 (0.07)	2 (0.01)	4 (0.06)	1 (0.01)
CHD	10 (0.14)	12 (0.09)	2 (0.03)	2 (0.01)	1 (0.01)	2 (0.01)
Heart failure	0	0	0	0	0	0
Stroke	1 (0.01)	2 (0.01)	0	1 (0.01)	1 (0.01)	1 (0.01)

Concordance: Both twins have the OA outcome in question, and both twins have the CVD outcome in question, i.e. they are similar for the outcomes in question both within- and cross-twin. OA: osteoarthritis, CVDs: cardiovascular diseases, card. arr: cardiac arrythmia, CHD: coronary heart disease, MZ: monozygotic DZ: dizygotic. All MZ twins: N=7017. All DZ twins: N=13 924.

S-Table 8. Correlations across traits, among pairs in which both twins survived through the entire follow-up period.

		Covariance of OA and CVDs in same individual	Covariance of OA and CVDs across twins in an MZ pair	Covariance of OA and CVDs across twins in an DZ pair	Indication of covariation explained by genetics	Indication of covariation explained by unique env. Factors
		Within-twin cross-trait, rPT (95% CI)	Cross-twin cross-trait rMZ (95% CI)	Cross-twin cross-trait rMZ (95% CI)	Non-overlapping 95% CIs, rMZ vs rDZ	Non-overlapping 95% CIs, rPT vs rMZ
Any of CVDs	Crude	0.22 (0.19-0.24)	0.27 (0.24-0.31)	0.16 (0.14-0.18)	Yes	Yes
	Adj.*	0.07 (0.04-0.09)	0.08 (0.05-0.12)	0.04 (0.02-0.06)	No	No

rPT: phenotypic correlation, rMZ: correlation in monozygotic twins, rDZ: correlation in dizygotic twins, OA: osteoarthritis, CVDs: cardiovascular diseases, card. arr: cardiac arrythmia, CHD: coronary heart disease. *Adjusted for age and sex.

S-Methods 1 – Computer code

```
##bivariate ACE model with two binary outcomes as presented in Magnusson et al., 2024:

#"Shared genetic factors between osteoarthritis and cardiovascular disease may underlie common etiology"


#Software dependencies and operating systems

#RStudio v2023.06.2, OpenMX: Extended Structural Equation Modelling v2.21.11

#typicall install time: 10 minutes

#Data cannot be shared for privacy reasons however are available upon request to authorized researchers at

#https://ki.se/en/research/swedish-twin-registry-for-researchers

#Expected run time: 4 hours


#Computer code

library(OpenMx)

###---------------part I data---

rm(list=ls())

setwd("....")


#################


Bivdata <- read.table ('....txt', header=T, sep="\t")

names(Bivdata)

mxOption(NULL, "Number of Threads", omxDetectCores()-1)


#select vars indicating any site OA, any of severe CVDs, and covariates age, sex and BMI, all vars for twin 1 and twin 2, respectively

vars		<-c('diOA','diCVDall')

selVars <-c('diOA1', 'diCVDall1', 'diOA2', 'diCVDall2' )

useVars		<-c('diOA1', 'diCVDall1', 'diOA2', 'diCVDall2','age1','age2', 'sex1', 'sex2', 'bmi1', 'bmi2')


#declare data to be binary

Bivdata$diOA1		<-mxFactor(Bivdata$diOA1, levels=c(0:1) )

Bivdata$diOA2		<-mxFactor(Bivdata$diOA2, levels=c(0:1) )

Bivdata$diCVDall1	<-mxFactor(Bivdata$diCVDall1, levels=c(0:1) )

Bivdata$diCVDall2	<-mxFactor(Bivdata$diCVDall2, levels=c(0:1) )
```

```
###########################################################################################
################################

#Select Data for Analysis

Bivdata$sex1    <-mxFactor(Bivdata$sex1, levels=c(0:1) )

Bivdata$sex2    <-mxFactor(Bivdata$sex2, levels=c(0:1) )

mzData <- subset(Bivdata, zygo2==1, useVars)

dzData <- subset(Bivdata, zygo2==0, useVars)


###---------------------------------------------------------------------------part II thresholds

nv       <- 2              # number of variables per twin

ntv      <- nv*2 # number of variables per pair

nth      <- 1              # number of max thresholds (we have only 1 threshold, because binary outcomes)


# CREATE LABELS & START VALUES as objects(to ease specification)

ThPat           <-c(T,T) #this means that the second threshold is false for both variables

StTH            <-c(1, 1 )


#Specify ACE Model, with ONE overall set of Thresholds without Age effects on thresholds

laLower <- function(la,nv) { paste(la,rev(nv+1-sequence(1:nv)),rep(1:nv,nv:1),sep="_") }

LabTh           <-c('T_11','T_12')


obsAge1         <- mxMatrix( type="Full", nrow=1, ncol=1, free=F, labels=c("data.age1"), name="Age1")

obsAge2         <- mxMatrix( type="Full", nrow=1, ncol=1, free=F, labels=c("data.age2"), name="Age2")

obssex1<- mxMatrix( type="Full", nrow=1, ncol=1, free=F, labels=c("data.sex1"), name="sex1")

obssex2<- mxMatrix( type="Full", nrow=1, ncol=1, free=F, labels=c("data.sex2"), name="sex2")

obsbmi1         <- mxMatrix( type="Full", nrow=1, ncol=1, free=F, labels=c("data.bmi1"), name="bmi1")

obsbmi2         <- mxMatrix( type="Full", nrow=1, ncol=1, free=F, labels=c("data.bmi2"), name="bmi2")


# Matrix & Algebra for expected means (SND), Thresholds

Mean            <-mxMatrix( type="Zero", nrow=1, ncol=ntv, name="Mean" )

Tr              <-mxMatrix( type="Full", nrow=nth, ncol=nv, free=ThPat, values=StTH, labels=LabTh,
lbound=c(-3,.001), ubound=4, name="Th")

inc             <-mxMatrix( type="Lower",nrow=nth, ncol=nth, free=F, values=1, name="Low")


LabCov <-c('BageThiq', 'BageThadhd')
```

betaA <-mxMatrix(type="Full", nrow=nth, ncol=nv, free=T, values=.2, labels=LabCov, name="BageTH")

LabCovSex <-c('BsexThiq', 'BsexThadhd')

betaS <-mxMatrix(type="Full", nrow=nth, ncol=nv, free=T, values=.2, labels=LabCovSex, name="BsexTH")

LabCovbmi <-c('BbmiThiq', 'BbmiThadhd')

betabmi <-mxMatrix(type="Full", nrow=nth, ncol=nv, free=T, values=.2, labels=LabCovSex, name="BbmiTH")

Thres <-mxAlgebra(expression= cbind(Low%*%Th + BageTH%x%Age1 + BsexTH%x%sex1 + BbmiTH%x%bmi1,

Low%*%Th + BageTH%x%Age2 + BsexTH%x%sex2 + BbmiTH%x%bmi2), name="expThres")

#---------------------part III ACE model

##we can take the starting values from the data (per https://openmx.ssri.psu.edu//node/4224), although the example is univariate

#define paths

pathA <- mxMatrix(type="Lower", nrow=nv, ncol=nv, free=TRUE, values=c(0.1,-0.05273827,0.1), label=laLower("a",nv), name="a")

pathC <- mxMatrix(type="Lower", nrow=nv, ncol=nv, free=TRUE, values=c(0.1,0.1,0.1), label=laLower("c",nv), name="c")

pathE <- mxMatrix(type="Lower", nrow=nv, ncol=nv, free=TRUE, values=c(0.1,0.04382757,0.1), label=laLower("e",nv), name="e")

Matrices generated to hold A, C, and E components and total Variance

covA <- mxAlgebra(expression=a %*% t(a), name="A")

covC <- mxAlgebra(expression=c %*% t(c), name="C")

covE <- mxAlgebra(expression=e %*% t(e), name="E")

covP <- mxAlgebra(expression=A+C+E, name="V")

Algebra for expected Variance/Covariance Matrices in MZ & DZ twins

covMZ <- mxAlgebra(expression= rbind(cbind(A+C+E , A+C),

cbind(A+C , A+C+E)), name="expCovMZ")

covDZ <- mxAlgebra(expression= rbind(cbind(A+C+E , 0.5%x%A+C),

cbind(0.5%x%A+C , A+C+E)), name="expCovDZ")

Algebra to compute standardized variance components

covP <- mxAlgebra(expression=A+C+E, name="V")

StA <- mxAlgebra(expression=A/V, name="h2")

StC <- mxAlgebra(expression=C/V, name="c2")

```
StE      <- mxAlgebra( expression=E/V, name="e2")


# Algebra to compute Phenotypic, A, C & E correlations

matI     <- mxMatrix( type="Iden", nrow=nv, ncol=nv, name="I")

rph      <- mxAlgebra( expression= solve(sqrt(I*V)) %*% V %*% solve(sqrt(I*V)), name="Rph")

rA       <- mxAlgebra( expression= solve(sqrt(I*A)) %*% A %*% solve(sqrt(I*A)), name="Ra" )

rC       <- mxAlgebra( expression= solve(sqrt(I*C)) %*% C %*% solve(sqrt(I*C)), name="Rc" )

rE       <- mxAlgebra( expression= solve(sqrt(I*E)) %*% E %*% solve(sqrt(I*E)), name="Re" )


# Constraint on total variance of Ordinal variables (A+C+E=1)

mUnv   <- mxMatrix( type="Unit", nrow=nv, ncol=1, name="Unv" )

varL     <- mxConstraint( expression=diag2vec(V)==Unv, name="VarL" )




# Algebra to compute Rph-A, Rph-C & Rph-E between two diseases

rphace<- mxAlgebra( expression= cbind (          (sqrt(h2[1,1])*Ra[2,1]*sqrt(h2[2,2])),

                              (sqrt(c2[1,1])*Rc[2,1]*sqrt(c2[2,2])),

                              (sqrt(e2[1,1])*Re[2,1]*sqrt(e2[2,2])) ), name="RphACE" )

# Data objects for Multiple Groups

dataMZ   <- mxData( observed=mzData, type="raw" )

dataDZ   <- mxData( observed=dzData, type="raw" )


# Objective objects for Multiple Groups

objMZ   <- mxExpectationNormal( covariance="expCovMZ", means="Mean", dimnames=selVars,
thresholds="expThres" )

objDZ   <- mxExpectationNormal( covariance="expCovDZ", means="Mean", dimnames=selVars,
thresholds="expThres" )



fitFunction <- mxFitFunctionML()



# Combine Groups

pars              <- list(obsAge1, obsAge2, obssex1, obssex2, obsbmi1, obsbmi2, Mean, betaA, betaS,
betabmi, Tr, Thres, pathA, pathC, pathE, covA, covC, covE, covP, StA, StC, StE, matI, rph, rA, rC, rE,
rphace, mUnv, varL, inc )

modelMZ                 <- mxModel( pars, covMZ, dataMZ, objMZ, fitFunction, name="MZ" )

modelDZ                 <- mxModel( pars, covDZ, dataDZ, objDZ, fitFunction, name="DZ" )

minus2ll          <- mxAlgebra( expression=MZ.objective + DZ.objective, name="m2LL" )
```

```
obj                 <- mxFitFunctionAlgebra( "m2LL" )
Conf1               <- mxCI (c ("MZ.h2","MZ.e2","MZ.c2") )
Conf2               <- mxCI (c ("MZ.Rph[2,1]") )
Conf3               <- mxCI (c ("MZ.RphACE[1,1]", "MZ.RphACE[1,2]", "MZ.RphACE[1,3]") )
Conf4   <- mxCI (c ('MZ.expCovMZ[2,1]', 'MZ.expCovMZ[1,4]', 'DZ.expCovDZ[1,4]','DZ.expCovDZ[2,1]'))
AceModel2       <- mxModel( "ACE", modelMZ, modelDZ, minus2ll, obj, Conf1, Conf2, Conf3, Conf4)
# 4) RUN AceModel
AceFit2   <- mxTryHardOrdinal(AceModel2, intervals=TRUE, silent=F, OKstatuscodes=c(0,1),extraTries = 4)
#if the above converged, just run again to get Cis
#AceFitCI<-mxRun(AceFit, intervals=TRUE)
(AceSumm  <- summary(AceFit2))

#AceFit2<-mxRun(AceFit, intervals=TRUE)

#to save a summary of correlations and model fit
write.table(AceSumm$CI,file="...csv", sep="\t" )
save.image("...RData")

#to obtain parameter estimates in S-Figure 1:
AceFit2$output$algebras

# Start values from best fit in small dataset:
AceFitCI$output$estimate
#status code from the solution
AceFit$output$status$code
```

S-Methods 2 – Computer code

##Cox regression analysis of cumulative concordance for OA and severe CVD, as presented in Magnusson et al., 2024:

#"Shared genetic factors between osteoarthritis and cardiovascular disease may underlie common etiology"

#Software dependencies and operating systems

#StataNow/MP 18.5 for Windows (64-bit x86-64)

#typicall install time: 30 minutes

#Data cannot be shared for privacy reasons however are available upon request to authorized researchers at

#https://ki.se/en/research/swedish-twin-registry-for-researchers

#Expected run time: 1 minute

#Computer code

*load data

use ..., clear

*calculate time

gen years = dateofconc_forOA_CVD - jan1_1997

*declare data to be time to event data, with the date of final twin having either OA or CVD as the last event being the exit date

stset years, failure(outcome==1), id(LopNrTw1)

*graph the cumulative concordance

sts graph, failure by(zygo) risktable(, failevents order(1 "MZ twins" 2 "DZ twins" size(small) title(, size(small))) ci title("", size(small)) xtitle("Years from start of followup", size()) ytitle(Cumulative Incidence of concordance for OA and CVD", size(small)) xlabel(, labsize(small)) ylabel(0(0.005)0.015, labsize(small)) legend(ring(0) position(2) rows(2) size(small))

*recode covariate data to indicate both health risk and degree of shared environment

*example of one of binary data

gen mean_smoke = (eversmokerTw1 + eversmoker Tw2)/2

*recode 0 to indicate shared for no risk factor, 0.5 to indicate discordance for risk factor, 1 to indicate both have risk factor

*include these as factor variables in analyses (i.)

*univariate cox regression analyses

```
foreach x of varlist zygo zygsex mean_educ mean_obesity mean_smoke mean_physact {
stcox i.`x'
}

stcox age1997

*multivariate cox regresion analyses (omit physical activity due to missing data)
stcox i.zygsex age1997 i.mean_educ i.mean_smoke i.mean_obesity
```
