# Supplementary material for: Shared genetic factors between osteoarthritis and cardiovascular disease may underlie common etiology
Source: Nat Commun. 2024 Nov 6;15:9569. doi: 10.1038/s41467-024-53812-2 (PMC11538479; doi:10.1038/s41467-024-53812-2)
Supplement: Supplementary file 2 — Reporting summary [file 41467_2024_53812_MOESM2_ESM.pdf]

nature portfolio

Corresponding author(s): Karin Magnusson

Last updated by author(s): Sep 19, 2024

Reporting Summary

Nature Portfolio wishes to improve the reproducibility of the work that we publish. This form provides structure for consistency and transparency in reporting. For further information on Nature Portfolio policies, see our Editorial Policies and the Editorial Policy Checklist.

Statistics

For all statistical analyses, confirm that the following items are present in the figure legend, table legend, main text, or Methods section.

n/a	Confirmed	
☐	☒	The exact sample size (*n*) for each experimental group/condition, given as a discrete number and unit of measurement
☐	☒	A statement on whether measurements were taken from distinct samples or whether the same sample was measured repeatedly
☒	☐	The statistical test(s) used AND whether they are one- or two-sided<br>*Only common tests should be described solely by name; describe more complex techniques in the Methods section.*
☐	☒	A description of all covariates tested
☐	☒	A description of any assumptions or corrections, such as tests of normality and adjustment for multiple comparisons
☐	☒	A full description of the statistical parameters including central tendency (e.g. means) or other basic estimates (e.g. regression coefficient) AND variation (e.g. standard deviation) or associated estimates of uncertainty (e.g. confidence intervals)
☒	☐	For null hypothesis testing, the test statistic (e.g. *F*, *t*, *r*) with confidence intervals, effect sizes, degrees of freedom and *P* value noted<br>*Give P values as exact values whenever suitable.*
☒	☐	For Bayesian analysis, information on the choice of priors and Markov chain Monte Carlo settings
☒	☐	For hierarchical and complex designs, identification of the appropriate level for tests and full reporting of outcomes
☒	☐	Estimates of effect sizes (e.g. Cohen's *d*, Pearson's *r*), indicating how they were calculated

Our web collection on statistics for biologists contains articles on many of the points above.

Software and code

Policy information about availability of computer code

Data collection	Methods of data collection varied with technological development from 1963 until study end. They also varied by the different data sources (i.e. Swedish Twin Registry, National Patient Register etc). Data management in the current study was performed using StataNow/MP 18.5 for Windows (64-bit x86-64)
Data analysis	RStudio v2023.06.2, OpenMX: Extended Structural Equation Modelling v2.21.11 (15) were used for all bivariate twin model analyses. StataNow/MP 18.5 for Windows (64-bit x86-64) were used for describing data and for Cox regression analysis.

For manuscripts utilizing custom algorithms or software that are central to the research but not yet described in published literature, software must be made available to editors and reviewers. We strongly encourage code deposition in a community repository (e.g. GitHub). See the Nature Portfolio guidelines for submitting code & software for further information.

Data

Policy information about availability of data

All manuscripts must include a data availability statement. This statement should provide the following information, where applicable:

- Accession codes, unique identifiers, or web links for publicly available datasets
- A description of any restrictions on data availability
- For clinical datasets or third party data, please ensure that the statement adheres to our policy

The dataset of this study was the Swedish Twin Registry, which is a property of Karolinska Institutet, provided to the researchers through a restricted-access

agreement that prevents sharing the dataset with a third party or publicly. Individual-level data of patients included in this paper after de-identification are considered sensitive and will not be shared. However, the individual-level data in the Swedish Twin Register are accessible to authorized researchers after ethical approval and application to "https://ki.se/en/research/swedish-twin-registry-for-researchers" administered by Karolinska Institutet. Data requests may be sent using the web application available at the STR website. Source data are provided with this paper.

Research involving human participants, their data, or biological material

Policy information about studies with human participants or human data. See also policy information about sex, gender (identity/presentation), and sexual orientation and race, ethnicity and racism.

Reporting on sex and gender	We consistently refer to the term "sex" as a biological attribute. Participants could not change their sex to their preferred gender during the data collection or analysis. The main analysis includes and are representative for both sexes. Analyses stratified by sex are included.
Reporting on race, ethnicity, or other socially relevant groupings	N/A
Population characteristics	59 970 monozygotic and dizygotic twins who were registered in the Swedish Twin Registry were included. Their mean [SD] age was 48 [16] years at start of follow-up, 54% were females and they had a mean [SD] body mass index of 23.4 [6.2] kg/m2.
Recruitment	Participants were recruited over a long period using various methods as described for the Swedish Twin Registry: Magnusson PK, Almqvist C, Rahman I, Ganna A, Viktorin A, Walum H, et al. The Swedish Twin Registry: establishment of a biobank and other recent developments. Twin Res Hum Genet. 2013 Feb;16(1):317-29.<br>This paper is referred to in our manuscript.
Ethics oversight	The Ethical Review Board at Lund University, Sweden

Note that full information on the approval of the study protocol must also be provided in the manuscript.

Field-specific reporting

Please select the one below that is the best fit for your research. If you are not sure, read the appropriate sections before making your selection.

☒ Life sciences ☐ Behavioural & social sciences ☐ Ecological, evolutionary & environmental sciences

For a reference copy of the document with all sections, see nature.com/documents/nr-reporting-summary-flat.pdf

Life sciences study design

All studies must disclose on these points even when the disclosure is negative.

Sample size	The study is based on secondary analyses of data collected for various purposes. Thus, we could not do sample size calculations a priori. The sample size was obtained through inclusions and exclusions as described at p. 6.
Data exclusions	To be able to apply the classical bivariate twin design/model, where both twins in a pair can be observed for a minimum amount of time, we excluded 10 810 singletons and 9 960 twins in pairs in which one or both twins were dead at the start of follow-up or during the first year.
Replication	The contribution of genetic vs environmental factors have been assessed using two different analytical approaches: the bivariate twin model and Cox regression analyses modeling time until pair-wise concordance for our outcomes. For the bivariate twin model, we also included a sensitivity analysis.
Randomization	Not applicable because we did not test an intervention.
Blinding	Not applicable because we did not test an intervention.

Reporting for specific materials, systems and methods

We require information from authors about some types of materials, experimental systems and methods used in many studies. Here, indicate whether each material, system or method listed is relevant to your study. If you are not sure if a list item applies to your research, read the appropriate section before selecting a response.

Materials & experimental systems

n/a	Involved in the study
☒	☐ Antibodies
☒	☐ Eukaryotic cell lines
☒	☐ Palaeontology and archaeology
☒	☐ Animals and other organisms
☒	☐ Clinical data
☒	☐ Dual use research of concern
☒	☐ Plants

Methods

n/a	Involved in the study
☒	☐ ChIP-seq
☒	☐ Flow cytometry
☒	☐ MRI-based neuroimaging

Plants

Seed stocks	N/A
Novel plant genotypes	N/A
Authentication	N/A
